# Autoimmune protocol diet: A personalized elimination diet for patients with autoimmune diseases

**DOI:** 10.1016/j.metop.2024.100342

**Published:** 2024-12-30

**Authors:** Eleni C. Pardali, Arriana Gkouvi, Kalliopi K. Gkouskou, Anastasios C. Manolakis, Christina Tsigalou, Dimitrios G. Goulis, Dimitrios P. Bogdanos, Maria G. Grammatikopoulou

**Affiliations:** a Unit of Immunonutrition and Clinical Nutrition, Department of Rheumatology and Clinical Immunology, Faculty of Medicine, School of Health Sciences, University of Thessaly, Biopolis, Larissa, Greece; b Department of Biology, Medical School, National and Kapodistrian University of Athens, Goudi Campus, Athens, Greece; c GENOSOPHY P.C., Athens, Greece; d Department of Gastroenterology, General University Hospital of Larissa, Larissa, Greece; e Laboratory of Hygiene and Environmental Protection, Medical School, Democritus University of Thrace, University Hospital, Alexandroupolis, Greece; f Unit of Reproductive Endocrinology, 1st Department of Obstetrics and Gynecology, Medical School, Aristotle University of Thessaloniki, Thessaloniki, Greece

**Keywords:** Inflammatory bowel disease, Hashimoto thyroiditis, Rheumatoid arthritis, Celiac disease, Food allergy, IgG, Food antigen, Diet

## Abstract

The autoimmune protocol diet (AIP) is a personalized elimination diet that aims to determine and exclude the foods that might trigger immune responses, leading to inflammation and symptomatology associated with autoimmune diseases. Focusing on gut health and the importance of the gut microbiome in immune regulation and overall well-being, the AIP starts by eliminating foods that might create negative effects on the patients and continues by developing a personalized and tailored diet plan for them. This comprehensive approach aims to mitigate symptoms and improve quality of life of individuals with autoimmune conditions. This review presents and critically appraises current knowledge on the AIP protocol, highlight any oversights, and discuss findings from relevant clinical trials.

## Highlights

• The Autoimmune Protocol (AIP) is a diet designed to enhance immune regulation, promote gut health, and maintain hormonal balance.
• Proper implementation of the AIP requires expert guidance, and adverse events remain inadequately understood.
• Research on the AIP is highlighting the need for further clinical studies to support its application.

## Abbreviations

ADHD: Attention Deficit Hyperactivity Disorder
AIP: Autoimmune Protocol
anti-TPO: Anti-Thyroid Autoantibodies
BC: Before Christ
BMI: Body Mass Index
DAMP: Damage-Associated Molecular Pattern
fT3: Free Tri-Iodothyronine
fT4: Free Thyroxine
FODMAP: Fermentable Oligosaccharides, Disaccharides, Monosaccharides, and Polyols
HT: Hashimoto Thyroiditis
IBD: Inflammatory Bowel Disease
IEC: Intestinal Epithelial Cell
IgG: Immunoglobulin G
MSQ: Medical Symptoms Questionnaire
NSAID: Non-Steroidal Anti-Inflammatory Drug
PAMPs: Pathogen-Associated Molecular Patterns
PRRs: Pattern-Recognizing Receptors
QoL: Quality of Life
RA: Rheumatoid Arthritis
RAPID3: Routine Assessment of Patient Index Data 3
RMDs: Rheumatic and Musculoskeletal Diseases
SF-36: 36-Item Short Form Health Survey
TLRs: Toll-Like Receptors
TSH: Thyroid Stimulating Hormone

## 1 Introduction

Rheumatic and musculoskeletal diseases range from classical autoimmunity to adaptive immune responses and auto-inflammatory disorders characterized by innate immunity activation and inflammasomes [1]. A complex network of cytokines and receptors is involved in the inflammatory reaction in RMDs, with treatment including therapeutic antibodies targeting disease-specific molecules [2]. Autoimmunity is characterized by self-reactive adaptive immune components, exceeding the threshold of systemic immune protection. The breaking of self-antigen tolerance initiated an attack against the body's cells [3,4]. The burden of autoimmune diseases is on a constant rise [5,6], exhibiting however, a female predominance. The co-manifestation of multiple autoimmune disorders is being attributed to shared pathogenetic mechanisms and genetic predisposition.

Emerging evidence highlights the role of diet in improving quality of life (QoL) and disease-related symptoms [7]. Diet has been established to contribute to the primary and secondary prevention of autoimmune diseases, as nutrients may regulate inflammatory and immune pathways [8]. Attaining an “optimal” or “suboptimal” diet may influence several immune-related physiological responses. For instance, the Western diet has been linked to an increased risk of engaging autoimmunity [9], as it dysregulates the gut barrier, affecting gut homeostasis and immune response [10]. The Autoimmune Protocol Diet (AIP) [11], a strict extension of the Paleolithic (Paleo) diet framework in an elimination manner, gained attention for its postulated ability to mitigate inflammation and improve symptoms of autoimmune diseases.

The AIP is based on the penetration of food antigens due to a dysfunction in the gut barrier, leading to increased permeability (“leaky gut”) [12]. The latter has been implicated in dysmotility and various autoimmune disorders [13]. Paired with microbial dysbiosis and in the presence of genetic susceptibility, it can synergistically act as an environmental trigger for autoimmunity [13]. Bacterial antigens stimulate intestinal immune cells, generating autoreactive cells that enter the systemic circulation and target peripheral organs [14]. At the same time, bacterial antigens can be translocated systematically through lymphatic connections, leading to autoreactive cell formation [14]. However, the classification of microbiota as either “good” or “bad” in a binary manner is misleading, as it does not acknowledge the interaction between the patient's genetic profile and the pre-existing microbiota [15].

## 2 Literature research

A comprehensive search was performed on MEDLINE/PubMed, clinicaltrials.gov, and the gray literature on June 2024, for human studies on patients with autoimmune diseases, applying the AIP.

## 3 Principles of the AIP

**Fig. 1:**
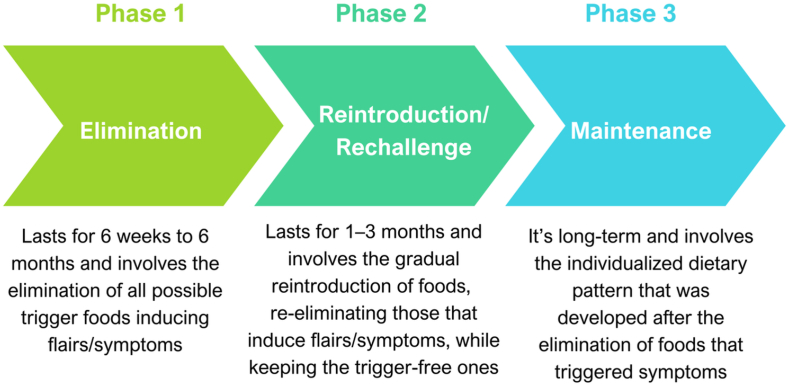
Phases of the AIP AIP: Autoimmune Protocol diet.

The AIP provides nutritious, whole foods, important for immune regulation, nutrient homeostasis, gut health, and hormone regulation [11]). It encompasses three phases: elimination, reintroduction, and maintenance (Fig. 1).

The gastrointestinal tract contains a series of protective layers that physically separate systemic circulation from external triggers, including mucus, epithelium, and lamina propria [16]. The intestinal epithelium layer consists of a monolayer of intestinal epithelial cells (IECs) connected by tight junction proteins and desmosomes, exercising strict control over permanent molecules [17]. This layer contains T-lymphocytes and innate lymphoid cells, whereas the lamina propria contains myeloid cells and lymphocytes [18,19]. IECs exhibit pattern-recognizing receptors (PRRs) such as toll-like receptors (TLRs) and identify pathogen-associated molecular patterns (PAMPs) or damage-associated molecular patterns (DAMPs) that can originate from food antigens or gut microbiota [20]. Subsequently, proinflammatory mediators, such as cytokines, are activated, leading to differentiation, maturation, and activation of immune system cells [21,22]. Macrophages and dendritic cells engulf molecules and interact with T-cells, leading to their differentiation specifically for this food antigen [23]. In food tolerance, T-cells differentiate into T-regulatory cells, which release anti-inflammatory cytokines and suppress immune responses. In food intolerance, T-cells differentiate into T-effector cells that may trigger a systemic immune response [24].

### 3.1 Unfolding the AIP

The AIP's rationale is to reduce inflammation, support gut health, and manage autoimmune conditions by eliminating foods that might trigger immune responses and exacerbate symptoms.

#### 3.1.1 Elimination phase

During the first phase, grains, legumes, nightshades, nuts, seeds, dairy, eggs, coffee, and alcohol are completely removed from the diet (Table 1). In addition, all refined sugars, oils, processed foods, food additives, artificial colors, and flavorings are excluded due to their contribution to gut dysfunction. The use of non-steroidal anti-inflammatory drugs (NSAIDs) is also avoided during this phase. Patients are encouraged to consume nutrient-dense whole foods, such as vegetables, fruits, mono- and poly-unsaturated fatty acids, tubers, wild game, poultry, organ, and non-processed meats (Table 1) [11]. Gluten-rich grains are avoided, as their glycoprotein extract, gliadin, is implicated in autoimmunity [25]. This phase spans from 6 weeks to 6 months [11]. This prolonged duration stems from plasma B-cells living from a few days to a few months, while immunoglobin G (IgG) antibodies (mostly associated with immune tolerance [26]) have a half-life of 21 days [27]. B-cells contribute to food allergen tolerance by producing allergen-specific IgG antibodies [11].

**Table 1:** AIP food list.

Foods to consume	Foods to avoid
Nose-to-tail grass-fed or wild-caught animal proteins, including meat (beef, bison, chicken, lamb, mutton, pork, turkey, wild game), fowl, organ meats, bone broth, heart, kidney liver, and tongue, fish (anchovies, catfish, cod, halibut, herring, mackerel, mahi, salmon, sardines, snapper, tilapia, trout, tuna), shellfish (clams, crab, crawfish, lobster, mussels, octopus, oysters, prawns, scallops, shrimp, squid)	All grains, eggs (and egg derivatives; mayo), and legumes, such as adzuki beans green beans, black beans, white beans, kidney beans, garbanzo beans, fava beans, peanuts, pinto beans, red beans, lupin, soy, lentils, peas, hummus
None	Barley, corn, durum, fonio, Job's tears, kamut, millet, oats, rice, rye, sorghum, spelled, teff, triticale, wheat, wild rice, crackers, rice waffles or pasta made with brown rice, teff, millet, quinoa, amaranth, tapioca, buckwheat, and potato flour
Coconut oil, milk and butter, olive oil, avocado, avocado oil/butter, black/green olives	All nuts and seeds (e.g., almonds, walnuts, cashews, sesame, flax seeds, hazelnuts, hemp seeds, macadamia nuts, pecans, pine nuts, pistachios, chia seeds, etc.), and their derivatives (e.g., canola oil, walnut oil, almond flour/milk, tahini)
Artichokes	Amaranth, buckwheat, chia, quinoa
Herbs and spices not derived from seeds, such as cinnamon, turmeric, thyme, oregano, basil, rosemary, turmeric, dill, ginger, sage, chives, basil, bay leaf, peppermint, garlic, cinnamon, thyme, parsley, saffron, mint	Anise, annatto, black caraway, celery seed, coriander, cumin, dill, fennel, fenugreek, mustard, nutmeg
Fresh fruits and vegetables; apples, apricots, asparagus, bananas, cantaloupes, capers, arrowroot sweet potatoes, greens, broccoli, brussels sprouts, lettuce, cucumbers, zucchini, cabbage, beets, cauliflower, carrots, collard greens, cassava (tapioca, kale yuca), celery, cherries, cucumbers, dates, figs, honeydew melons kohlrabi, jicama, mustard greens, pumpkins, cabbage, squash, radishes, rutabagas, kiwis, mangoes, nectarines, papayas, peaches, pears, pineapples, plantains, plums pomegranates, watermelons, zucchini, radicchio, turnips, taro, watercress, yams, berries, melon, okra, clementines, grapefruit, lemons, limes, mandarin oranges, oranges, chives, garlic, leeks, onions scallions shallots spring onions etc.	Ashwagandha, bell peppers (sweet peppers), cayenne peppers, cape gooseberries, eggplant, garden, goji berries, hot peppers (chili peppers), naranjillas, paprika, pepinos, pimentos, potatoes tamarillos, tomatillos, tomatoes, ketchup
Non-dairy fermented foods, including water-kefir, sauerkraut, kombucha, pickled veggies, apple cider vinegar, red wine vinegar, and kimchi	All dairy products, including yogurt, ghee butter, kefir, milk, cheese, and cream
Honey, maple syrup, date sugar, coconut sugar, molasses, dried fruits	Granulated sugar, added or artificial sweeteners (acesulfame potassium, aspartame, erythritol, mannitol, neotame, saccharin, sorbitol, stevia, sucralose, xylitol), sugar-sweetened beverages, sugary soda drinks, food additives, emulsifiers, food chemicals, agave, barley malt, barley malt syrup, brown rice syrup, brown sugar, cane crystals, cane sugar (refined), caramel, corn syrup, crystalline fructose, fructose, fruit juice, fruit juice concentrate, galactose, glucose, glucose solids, golden syrup, high- fructose corn syrup, invert sugar, inulin, lactose, malt syrup, maltodextrin, maltose
Herbal teas, green tea, chamomile	Alcohol, beer, wine, and coffee

AIP: Autoimmune protocol diet.

#### 3.1.2 Reintroduction phase

During the second phase, eliminated foods are reintroduced to identify the ones that trigger individual responses. Generally, there is no rule of thumb on how to initiate the reintroduction. The most common manner is to reintroduce the foods that each patient enjoys the most, or the ones that are less likely to induce negative responses, in an effort to increase the food options. With this in mind, foods have been categorized into four groups based on their likelihood of being well-tolerated [11]. Group 1 consists of egg yolks, legumes, seed oils, and nut oils [11]. Group 2 includes nuts and seeds, cocoa, egg whites, and alcohol [11]. Group 3 comprises cashews and pistachios, eggplant, coffee, and fermented dairy, while Group 4 includes all dairy, white rice, nightshades, alcohol, and gluten-free grains [11]. This phase is time-consuming; if performed methodically however, it results in distinct beneficial health outcomes for each individual.

#### 3.1.3 Maintenance phase

The last AIP phase involves maintaining the protocol and has no specific duration. It aims to provide a healthy diet and lifestyle that will reduce autoimmune responses. In this manner, each patient adopts a dietary pattern associated with a lack of intolerances, considering the reintroduction phase responses.

### 3.2 Going beyond the paleo diet

The Paleo diet originated in the Paleolithic age, at the time of the primitive *Homo sapiens*, who relied mainly on hunting for food [28]. Thus, the Paleo diet was based on grass-fed, pasture-raised and game meat, small fish, eggs, fruit, leafy vegetables, nuts, and seeds, according to the geographic region [29]. It consists of a whole-food diet, with nutritionally dense foods of high quality. The Paleo diet is potentially beneficial to metabolic syndrome [30], cardiovascular diseases [31], cancer [32], insulin resistance [33], type 2 diabetes [34], and body composition [35]. Several foods are eliminated during its implementation, including processed foods, added sugars (except for honey), artificial sweeteners, grains, dairy, and legumes [36]. Thus, the Paleo diet consists of a specific, horizontal dietary pattern, similar for each patient. On the contrary, the AIP is personalized, with different foods allowed or eliminated, depending on individual tolerance. Both diet patterns include an elimination phase, but the AIP goes a step beyond the Paleo diet, aiming to minimize the existence of any possible antigen that might trigger an autoimmune response [11,37]. Thus, individuals who follow the AIP will develop a personalized dietary pattern based on their food tolerances. Moreover, accurately implementation of the AIP requires guidance and support, as it is crucial to correctly follow the phases to address individual food sensitivities and health concerns. In contrast, the Paleo diet does not require this level of expert guidance and assistance. Furthermore, the AIP elimination phase is stricter, with more foods being excluded than the Paleo diet, including eggs, nuts and seeds, nightshades, food additives, refined oils, coffee, and alcohol [11].

### 3.3 The elimination timeline

**Fig. 2:**
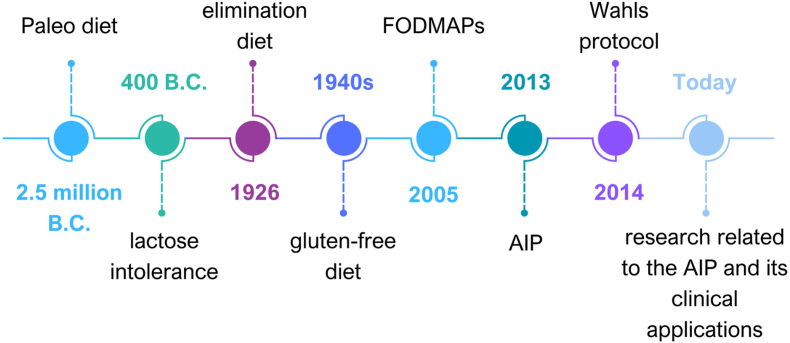
Timeline of the AIP development AIP: Autoimmune Protocol Diet, B.C.: Before Christ, FODMAPs: Fermentable Oligosaccharides, Disaccharides, Monosaccharides, and Polyols.

The basis for elimination diets dates back (Fig. 2) to the Paleolithic era due to the need to search for food and the limited options available at different times [28]. The subsequent reference to food elimination was made by Hippocrates in 400 BC, referring to lactose intolerance, suggesting eliminating dairy products to relieve discomfort [38]. The term “elimination diets” was first reported in 1926 by Dr. Rowe in 1944 [39]. During the same period, the elimination of gluten among patients with celiac disease was reported [40]. In the early 2000s, the fermentable oligosaccharides, disaccharides, monosaccharides, and polyols (FODMAP) diet emerged as another iteration of elimination diets recommended for alleviating symptoms of irritable bowel syndrome [41]. Over the past decade, specific forms of elimination diets, including the AIP and the Wahls protocol [42], appeared and were recommended for autoimmune diseases and multiple sclerosis, respectively. There is a concerted effort and need to perform clinical studies to ascertain AIP's clinical efficacy accurately.

## 4 Clinical insights

### 4.1 Evidence on AIP applications

Elimination diets have long been used to manage diseases, including celiac disease, allergies, and inflammatory bowel disease (IBD). Regarding autoimmune diseases, the AIP diet has been implemented in organ-specific and systemic autoimmune diseases such as Hashimoto thyroiditis (HT), IBD and rheumatoid arthritis (RA), improving QoL and disease-related symptoms [[43], [44], [45]]. Primary studies implementing the AIP in autoimmune diseases are presented in Table 2.

**Table 2:** Primary studies implementing the AIP.

First author	Origin	Design	Diagnosis	Participants	Intervention(s)	Intervention duration	Outcomes	Results
Abbott [44]	USA	UCT	HT	N = 16, women with overweight/normal BW and HT, age 20–45 years	Online health coaching on a phased elimination diet (AIP)	10 wk	QoL (SF-36) and clinical symptom burden (MSQ), anthropometry (BW, BMI), thyroid function (TSH, fT_3_, fT_4_, TGA, TPO), inflammation (hs-CRP), CBC	Improvement in QoL, regarding the physical role functioning, emotional role functioning, vitality, mental health, social role functioning, bodily pain and general health subscales. The clinical symptom burden decreased post-intervention. No changes in thyroid function. Inflammation (hs-CRP) decreased. BMI and BW decreased.
Ihnatowicz [43]	Poland	UCT	HT	N = 28, adults with HT (including 1 male)	AIP	12 wk	Dietary intake, anthropometry, HT symptoms, thyroid function (TSH, fT_3_, fT_4_, TPO, TGA, thyroid US)	Following the AIP, the number of people reporting symptoms of malaise decreased, fT_3_ and fT_4_ concentrations decreased, remaining within reference range. Slight reduction in TSH and TGA; TPO showed an upward trend. Decrease in thyroid gland volume. Decrease in BW and muscle mass.
Konijeti [[45], [46], [47]]	USA	UCT (NCT03-512,327)	IBD (UC and CD)	N = 15, patients with IBD, erosions on endoscopy and/or elevated FC, mean disease duration 19 years, active biologic use in 47 %	AIP	6 wk AIP, followed by a 5-wk maintenance phase	CRP, partial Mayo score, HBI, FC, mucosal RNA expression (RNA-seq on colonic tissue biopsy), endoscopic picture (SES-CD, Rutgeerts and Mayo endoscopy subscore)	Nutrient repletion was initiated for deficiencies in vitamin D (*n* = 3) and Fe (*n* = 6). Clinical remission in 73 % by wk 6, maintained during maintenance phase. In UC, partial Mayo score improved from 5.8 to 1.2 (wk 6) and 1.0 (wk 11). In CD, HBI improved from 6.7 to 3.3 (wk 6) and 3.4 (wk 11). CRP and FC did not change. In patients with follow-up endoscopy (wk 11), improvements in Mayo endoscopic (*n* = 4), SES-CD (*n* = 1), and Rutgeerts score (*n* = 1). Modulation of pathways involved in inflammation, metabolic processes, DNA repair, and cellular proliferation, indicating cellular responses to the AIP.
McNeill [48,49]	New Zealand	Cross-over, non-randomized	RA	N = 9, adults with RA	(i) AIP (ii) control: usual diet	12 wk AIP (i) 4 wk control (ii), 8 wk AIP (i)	QoL, RAPID3, RAID, fatigue, pain, sleep, emotional wellbeing	RAPID3 reduced from baseline to wk 12. On AIP, 4 patients reached a clinically meaningful reduction in RAPID3 of >1.2 and endpoint <2. Fatigue, sleep, and pain improved at wk 12.

AIP: Autoimmune protocol diet; BW: body weight; BMI; Body mass index; CBC: complete blood count; CD: Crohn's disease; CRP: C-reactive protein; DNA: deoxyribonucleic acid; FC: fecal calprotectin; fT_3_: free T_3_; fT_4_: free T_4_; HBI: Harvey Bradshaw Index; hs-CRP: high-sensitivity C-reactive protein; HT: Hashimoto's thyroiditis; IBD: inflammatory bowel disease; MSQ: Cleveland Clinic Center for Functional Medicine's Medical Symptoms Questionnaire; QoL: quality of life; RA: rheumatoid arthritis; RAID: Rheumatoid arthritis Impact of Disease; RNA: Ribonucleic acid; RAPID3: Routine Assessment of Patient Index Data 3; SES-CD: simple endoscopic score for Crohn's disease; SF-36: 36-Item Short Form Health Survey; TGA: anti-thyroglobulin antibodies; TPO: thyroid peroxidase antibodies; TSH: thyroid stimulating hormone; UC: ulcerative colitis; UCT: uncontrolled clinical trial; US: ultrasound, USA: United States of America, wk: week.

In HT, two uncontrolled clinical trials [43,44] concluded that patients undergoing the AIP improved and showed that the physical and emotional statuses had less malaise and enhanced general health subscales. In the first study, Field [47] studied women with HT who underwent a 10-week online AIP protocol; in the second [43], they implemented the AIP diet for 12 weeks. Both studies reported improvement in physical and emotional well-being, harnessing patient-reported outcome measures such as the 36-Item Short Form Health Survey (SF-36) and the Cleveland Clinic Center for Functional Medicine's Medical Symptoms Questionnaire (MSQ), as well as open-ended questions regarding malaise levels and disease-related symptoms. Objective findings, such as thyroid function, were assessed in both studies; the second [44] reported no changes, whereas the first reported decreased thyroid gland volume and lower free tri-iodothyronine (fT_3_) and free thyroxine (fT_4_) concentrations. At the same time, anti-thyroid autoantibodies (anti-TPO) increased, and thyroid stimulating hormone (TSH) concentrations decreased. Since fT_3_ and fT_4_ concentrations were reduced, while TSH and anti-TPO concentrations remained unchanged, adherence to the AIP may worsen thyroid function. Inflammatory markers improved significantly post-intervention [44]. Regarding the anthropometric indices, the first study [43] reported a decrease in body mass index (BMI), body fat (as a % of body weight) and muscle mass, indicating a calorie deficit. In contrast, the second [44] recorded a decrease in body weight and BMI. The same study [44] complemented the AIP with lifestyle modifications such as promoting support systems, sleep hygiene, stress management, movement, and increased time spent outdoors, which could have impacted the results.

In IBD, one uncontrolled trial [[45], [46], [47]] implemented the AIP in patients with active disease for 11 weeks. Clinical remission and endoscopic improvement were documented in most patients as opposed to inflammatory markers (C-reactive protein, fecal calprotectin), which remained unaltered. Based on further analysis, treatment with biological therapy did not affect outcomes. On the other hand, vitamin D and iron supplementation may have influenced the results.

In systemic autoimmune diseases, a non-randomized, crossover trial was conducted in patients with RA, comparing QoL measures following the AIP against the usual diet [48,49]. Preliminary results revealed improved sleep, fatigue, pain sensation, and improved patient-reported outcomes related to disease severity (Routine Assessment of Patient Index Data 3–RAPID3).

Table 3 illustrates the outcomes of the primary studies mentioned above. Adverse events reported in the studies above are listed in Table 4. The HT studies [43,44] had no moderate-to-severe adverse events, although some participants had mental challenges during the elimination phase. In the IBD trial [[45], [46], [47]], one participant suffered from partial small-bowel obstruction requiring hospitalization and another mentioned worsening symptoms and withdrew from the study.

**Table 3:** Reported adverse events associated with adherence to the AIP.

First author	AEs assessed	AEs reported
Abbott [44]	Nutrient deficiencies	Deficiencies in folate, vitamin B_12_, and B_2_
Ihnatowicz [43]	Any AE	None reported
Konijeti [[45], [46], [47]]	Partial small bowel obstruction, exacerbation of symptoms associated with an ileocecal valve stricture	1/9 patient with CD with an ileocecal valve stricture experienced symptom aggravation and was unable to finish the AIP 1/9 patients with CD with postoperative recurrence of ileal involvement experienced partial small bowel obstruction attributable to excessive intake of raw vegetables
McNeill [48,49]	Any AE	None reported

AE: adverse event; AIP: Autoimmune protocol diet; CD: Crohn's disease.

**Table 4:** Outcomes reported in studies evaluating the effects of the AIP in patients with autoimmune diseases.

First Author	Outcome category	Outcomes	Pre-AIP	Post-AIP	*p*-value
Abbott [44]	PRO (SF-36 subscales with score 0–100)^f^	Physical Functioning	80 (29)^i^	95 (10)^i^	0.0001
		Physical Role Functioning	25 (88)^i^	100 (50)^i^	0.001
		Emotional Role Functioning	33 (92)^i^	78 (19)^i^	0.0063
		Vitality	23 (19)^i^	58 (34)^i^	<0.0001
		Mental Health	54 (25)^i^	78 (19)^i^	<0.0001
		Social Role Functioning	63 (22)^i^	81 (22)^i^	0.0057
		Bodily Pain	68 (22)^i^	78 (21)^i^	0.0112
		General Health	40 (26)^i^	70 (35)^i^	<0.0001
	PRO (0–100)	MSQ^b^	92 ± 25^h^	29 ± 20^h^	<0.0001
	Inflammation	hs-CRP (mg/dL)	1.6 ± 1.7^h^	1.2 ± 1.3^h^	0.0219
		WBC (10^3^/μL)	5.6 ± 1.4^h^	5.1 ± 1.4^h^	NS
	Thyroid function	TSH (μIU/mL)	2.0 ± 1.5^h^	2.0 ± 1.4^h^	NS
		fT_3_ (pg/mL)	2.4 ± 0.6^h^	2.4 ± 0.5^h^	NS
		fT_4_ (ng/dL)	1.3 ± 0.4^h^	1.4 ± 0.4^h^	NS
		TPO (IU/mL)	225 ± 178^h^	219 ± 186^h^	NS
		TGA (IU/mL)	110 ± 261^h^	124 ± 293^h^	0.176
	Anthropometric	Body weight (lbs)	149.5 ± 19.5^h^	143.4 ± 16.7^h^	0.002
		BMI (kg/m^2^)	24.9 ± 2.6^h^	23.9 ± 2.2^h^	0.002
Ihnatowicz [43]	Thyroid function	TSH (mIU/L)	3.7 ± 4.1^h^	2.7 ± 21.6^h^	NS
		fT3 (pmol/L)	3.3 ± 0.4^h^	2.9 ± 0.8^h^	0.009
		fT4 (ng/dL)	1.4 ± 0.3^h^	1.2 ± 0.3^h^	0.044
		Anti-TPO (IU/mL)	210 ± 204^h^	294 ± 301^h^	NS
		Anti-TG (IU/mL)	317 ± 272^h^	301 ± 220^h^	NS
	Thyroid US	pp-usg (mL)	7.2 ± 3.9^h^	6.8 ± 3.2^h^	0.020
		Pt-usg (mL)	5.3 ± 2.7^h^	5.0 ± 2.3^h^	0.014
	Anthropometric	Body weight (kg)	69.0 ± 12.1^h^	65.5 ± 10.8^h^	<0.0001
		BMI (kg/m^2^)	25.6 ± 369^h^	24.3 ± 3.1^h^	<0.0001
		Body fat (% weight)	33.0 ± 7.6^h^	29.4 ± 8.0^h^	<0.0001
		Body muscle (% weight)	23.0 ± 8.0^h^	21.0 ± 5.0^h^	0.032
Konijeti [[45], [46], [47]]	Disease activity (CD)	HBI (1–25)^a^	6.7 ± 1.5^h^	3.4 ± 2.6^h^	0.004
		Abdominal pain (0–3)	0.6 ± 0.5^h^	0.6 ± 0.8^h^	NS
		Bowel movement (*n* of liquid/soft stools)	3.4 ± 2.2^h^	2.4 ± 1.3^h^	NS
		General well-being (0–4)	1.6 ± 0.5^h^	0.3 ± 0.8^h^	0.022
	Disease activity (UC)	Partial Mayo score (0–9)^c^	5.8 ± 1.2^h^	1.0 ± 2.0^h^	0.007
		Stool frequency (0–4)	2.0 ± 0.9^h^	0.2 ± 0.4^h^	0.012
		Rectal Bleeding (score 0–4)	1.8 ± 0.8^h^	0.3 ± 0.8^h^	0.017
	Quality of life (UC)	PGA (score 0–10)^d^	2.0 ± 0^h^	0.5 ± 0.8^h^	0.007
		SIBDQ (score 10–70)^g^	46.5 ± 12.5^h^	60.5 ± 4.8^h^	0.045
	Inflammation	Fecal calprotectin (μg/g)	471 ± 562^h^	112 ± 104^h^	NS
		CRP (mg/L)	3.9 ± 5.2^h^	3.4 ± 5.3^h^	NS
McNeill [48,49]	PRO	RAPID3 (0–30)^e^	2.5 [0.7–5.3]^j^	1.0 [0–2.5]^j^	NR
		Fatigue (VAS 0–10)	4.4 [0–10]^j^	1.1 [0–4]^j^	NR
		Sleep (VAS 0–10)	3.8 [0–10]^j^	1.2 [0–2]^j^	NR
		Pain (VAS 0–10)	3.3 [0–4.5]^j^	1.4 [0–3.5]^j^	NR

μL: microliter; μΙU: micro-international units; BMI; Body mass index; CBC: complete blood count; CD: Crohn's disease; CRP: C-reactive protein; dL: deciliter; fT_3_: free T_3_; fT_4_: freeT_4_; g: gram; HBI: Harvey Bradshaw Index; hs-CRP: high-sensitivity C-reactive protein; IU: international units; kg: kilogram; L: Liter; lbs: libra; mg: milligram; mIU: milli-international units; mL: milliliter; MSQ: Cleveland Clinic Center for Functional Medicine's Medical Symptoms Questionnaire; ng: nanogram; NR: not reported; NS: not significant; pg: picogram; PGA: Physician global assessment; pmol: picomole; PRO: Patient-reported outcome; RAPID3: Routine Assessment of Patient Index Data 3; SF-36: 36-Item Short Form Health Survey; TGA: anti-thyroglobulin antibodies; TPO: thyroid peroxidase antibodies; TSH: thyroid stimulating hormone; UC: ulcerative colitis; US: ultrasound; VAS: visual analogue scale; WBC: White blood cells; wk: week.

a HBI scores from 1 to 25: <5 remissions; 5–7 mild activity; 8–16 moderate activity; >16 severe activity and includes abdominal pain, bowel movement, and general well-being.

b MSQ scores from 0 to 100: Optimal score: less than 10; Mild toxicity: 10–50; Moderate toxicity: 50–100; Severe toxicity: over 100.

c Partial Mayo Score scores from 0 to 9: <2 remission; 2–4 mild activity; 5–7 moderate activity; >7 severe activity and includes stool frequency and rectal bleeding.

d PGA scores from 0, defined as the absence of disease activity, to 10, defined as maximum disease activity.

e RAPID-3 scores from 0 to 30: scores >12 high disease activity; > 6–12 moderate disease activity; > 3–6 low disease activity, and ≤3 remission.

f SF-36 scores are between 0 and 100 with 100 representing the highest level of functioning possible.

g SIBDQ scores from 0 to 70; 10–45 severely impaired QoL; 45–60 moderately impaired QoL; 60–70 slightly impaired QoL.

h Mean ± SD.

i Median (IQR).

j Mean [range].

### 4.2 Nutrient supplementation in AIP

Despite its stringent nature, the AIP advocates a nutritious regimen, rich in whole foods, including colorful vegetables and fruits, organic meat, and lean fish (Table 1). This approach ensures that individuals consume more nutrient-dense foods, possibly leading to greater vitamin and nutrient intakes than when on a standard Western diet. Despite the absence of extensive clinical studies examining possible nutrient deficiencies, the data focusing on specific vitamin deficiencies [44,46] are contentious. While the AIP includes some sources of meat and vegetables, excluding grains might result in deficiencies in vitamins B_1_, B_2_ and B_3_, fiber and iron [50]. The study on women with HT [44] revealed that 50 % of the participants exhibited folate, vitamin B_12_, or riboflavin deficiencies. Moreover, the Paleo diet may hinder adequate vitamin D and calcium intake [51], given the exclusion of vitamin D and calcium-rich dairy products [52,53]. Of course, the degree of nutrient deficiencies greatly depends on how many foods are excluded from an individual's diet, with more strict patterns offering less variety in the nutrient intake and, thus, a greater chance for nutrient deficiencies. Therefore, it would be false to state that all patients following the AIP have the same chances of developing nutrient deficiencies. Nonetheless, there is a need for comprehensive and well-designed studies to ascertain whether the implementation of the AΙP results in deficiencies, or if it necessitates adjuvant oral nutrient supplementation.

### 4.3 AIP limitations

The AIP research has limitations, such as adverse events (Table 4). In the study among patients with IBD [[45], [46], [47]], one patient with an ileocecal valve stricture experienced symptom aggravation and failed to complete the protocol. In the same protocol, a patient with postoperative recurrence of ileal Crohn's Disease required hospitalization due to partial small bowel obstruction that was caused by high consumption of raw vegetables, salads, and meat and lack of guidance by a registered dietitian [[45], [46], [47]]. Both patients had ileal strictures, sparking discussion regarding implementing AIP in patients with anatomic variations. As mentioned, nutrient deficiencies consist of a limitation when adhering to the AIP, as deficiencies in folate, vitamin B_12_, or riboflavin were apparent among women with HT [44], emphasizing the need for individualization and the implementation of a well-designed AIP plan delivered by a registered dietitian [44]. Notably, while some deficiencies were present at the study's onset, others were resolved by its conclusion. In addition, to implement the protocol correctly, the patient needs to be in full remission, as the basis of this diet regime is to detect immune responses based on the symptomatology [11]. Thus, if the reintroduction phase occurs while the patients are on a flare, they might be unable to determine the exact foods causing them dysfunction and, finally, observe a beneficial effect.

There is a lack of evidence to suggest the implementation of the AIP in populations with greater nutritional needs, such as pregnant and lactating women and children. In parallel, people with eating disorders or those who are prone to orthorexia may find the nature of the AIP restrictive and potentially triggering for disordered eating behaviors. Those with certain medical conditions and very specific guidelines regarding food intake are also warranted against following the AIP. People with allergies to foods allowed in the general AIP should aim for the additional exclusion of those prompting allergic reactions (IgG exclusion foods). Likewise, patients with malabsorption syndromes or ileal strictures should not follow the AIP due to its high fiber content.

Notably, some primary studies presented herein excluded patients with common comorbidities such as hypertension and did not allow for the intake of other medications, not allowing for external generalization (Table 5). An important limitation is that all studies are grossly underpowered, using subjective rather than objective measures. More studies are required to assess the effect of the AIP on the pharmacokinetics and pharmacodynamics of commonly used medications.

**Table 5:** Inclusion and exclusion criteria of the studies investigating the effects of AIP in patients with autoimmune diseases.

First author	Inclusion criteria	Exclusion criteria
Abbott [44]	HT diagnosis, English-speaking women, age 20–45 years, BMI 18.4–29.9 kg/m^2^	No definite diagnosis of HT, pregnancy, breastfeeding ≤ 6 months postpartum, hypertension, diabetes, heart disease, heart failure, liver failure, chronic renal disease, use of other medications (except FDA approved thyroid replacement medications), outside of pre-defined age and BMI limits
Ihnatowicz [43]	HT diagnosis, age 19–50 years, BMI >19 kg/m^2^	Pregnancy, breastfeeding, celiac disease, renal failure, liver failure, advanced atherosclerotic disease, malnutrition, eating disorders, use of an elimination diet the previous year, implanted electro-stimulators
Konijeti [[45], [46], [47]]	Symptomatic CD (defined as Harvey–Bradshaw index >5) or UC (partial Mayo clinic score >3), objective evidence of active disease (endoscopy or elevated fecal calprotectin), Facebook account, comfortable with e-mails	Pregnancy, breastfeeding, celiac disease, history of positive TTG antibodies, untreated infection, stoma or J-pouch, bowel surgery ≤ 12 wks before enrollment, tube or enteral feeding, elemental diet or parenteral alimentation ≤ 4 wks before initiation
McNeill [48,49]	Stable medication and supplements for >8 wks, unrestricted diet	NR

AIP: autoimmune protocol diet; BMI: Body mass index; CD: Crohn's disease; FDA: Food and Drug Administration; HT: Hashimoto's Thyroiditis; NR: not reported; TTG: anti-tissue transglutaminase; UC: ulcerative colitis; wks: weeks.

Apart from the concerns raised above, additional constraints exist regarding the AIP's implementation. People following the diet require comprehensive guidance to adopt a nutritionally balanced diet that avoids deficiencies. Furthermore, they will need assistance determining the duration of the elimination phase, beginning the reintroduction phase, sequencing the introduction of new foods, and how many days they should wait for the rechallenge. Many individuals report withdrawal symptoms like headaches, fatigue, irritability, or skin flare-ups, which may last for several days to a week, and they generally tend to feel worse before they feel better, as they might eliminate foods like caffeine, that might cause headaches or migraine in its absence. Additionally, the restrictive nature of the AIP may lead to feelings of social isolation and limitation, impacting individuals’ social lives. The AIP is not a dietary pattern that can be implemented for life; this is not its rationale. Remaining in the elimination phase can put an individual at risk for nutrient deficiencies and potential malnutrition. There are however, no long-term data on the AIP maintenance phase. Finally, there are no established guidelines or evidence to confirm adequacy in the protein intake.

### 4.4 Expanding the AIP role

Aside from incorporating the AIP into the management of autoimmune diseases, several other conditions may benefit from its application: individuals experiencing unexplained symptoms such as eczema and migraines, those seeking to gain a deeper understanding of their body, individuals aiming to enhance gut health and overall well-being, those looking to identify food allergies, sensitivities and intolerances, individuals with eosinophilic esophagitis [54], and people diagnosed with attention deficit hyperactivity disorder (ADHD) [55]. Despite the low evidence, several societies present AIP as a possible diet regimen for patients with autoimmune diseases like systemic lupus erythematosus [56].

## 5 Conclusion

Despite the limited clinical evidence, the AIP has been gaining popularity recently. It consists of a personalized elimination diet, with the rationale of reducing inflammation and alleviating symptoms associated with autoimmune diseases. In parallel, it promotes nutrient-rich, whole foods supporting gut health and supplying essential vitamins and minerals for immune and intestinal function and overall well-being. Although the AIP can be challenging, it is a customized and personalized diet plan that suits the patients’ individual needs, being a promising, powerful diagnostic and therapeutic tool.

Horizontal elimination diets like the FODMAP and the Paleo lack consistency in favorable outcomes across different populations [57]. On the other hand, the AIP has the potential to identify individual immune responses and tailor intake in a personalized approach. In this manner, it aligns with the contemporary call for customized nutrition. Whether the AIP will be the holy grail or the next fad diet for autoimmune diseases will depend on the research quantity and quality. Controlling autoimmunity with diet would be life-changing for patients. For this, a pledge for high-quality studies is made to elucidate the biological mechanisms of the AIP and expand the knowledge of its efficacy and safety.

## CRediT authorship contribution statement

**Eleni C. Pardali:** Writing – original draft, Visualization, Conceptualization. **Arriana Gkouvi:** Writing – original draft, Visualization, Investigation, Data curation. **Kalliopi K. Gkouskou:** Writing – review & editing. **Anastasios C. Manolakis:** Writing – review & editing, Investigation. **Christina Tsigalou:** Writing – review & editing, Project administration, Funding acquisition. **Dimitrios G. Goulis:** Writing – review & editing, Supervision, Methodology. **Dimitrios P. Bogdanos:** Writing – review & editing, Supervision, Methodology, Conceptualization. **Maria G. Grammatikopoulou:** Writing – review & editing, Writing – original draft, Supervision, Methodology, Data curation, Conceptualization.

## Funding

This review received no external funding.

## Conflicts of interest

None.
